# Reimagining global health architecture for a sustainable future

**DOI:** 10.1016/j.eclinm.2025.103532

**Published:** 2025-09-22

**Authors:** eClinicalMedicine

Global health architecture—the system of principles, agreements, and actors of global health—emerged in its modern form in the post-Second World War period that assumed steady economic expansion. Today, this growth-dependent model is in crisis; debt-driven instability undermines health expenditure, ecological limits are exceeded, and political distortion fuels militarisation and weakens multilateral institutions. The COVID-19 pandemic revealed serious shortcomings in coordination, financing, and leadership in global health—5 years on, these governance gaps remain unresolved. Global political polarisation has intensified, further weakening trust in multilateral institutions and straining under-resourced health systems. Addressing this vacuum in global health architecture requires more than institutional reform; it calls for a broader socioecological transformation. One Health, an integrated, unifying approach that aims to sustainably balance and optimise the health of people, animals, and ecosystems, has emerged as the conceptual pillar of this transformation. World Environmental Health Day, marked on Sept 26, 2025, and led by the International Federation of Environmental Health, focuses this year on clean air and serves as a reminder of the need to place sustainability and the integration of One Health in all policies to shape a new global health architecture.

The Lancet One Health Commission, launched in July 2025, frames health as a deeply ecological concept, emerging from the interplay among humans, animals, plants, and the environment. It calls for a socioecological transformation that rethinks food systems, economic models, and power structures. Central to this vision is the transition from extractive, industrial food production to resilient, equitable, and ecologically sound systems. The transformation is essential, but the path toward implementation requires a cautious approach. If not designed and implemented truly inclusively, policies meant to reduce environmental harm might inadvertently burden the very populations they aim to protect, such as smallholder farmers, low-income communities, and marginalised groups. There is also a risk that powerful corporate interests will adapt reforms in ways that shield their own practices while shifting costs downward onto groups with fewer resources, which in turn might exacerbate resource concentration among the ultra-rich and further widen the gap between them and the broader population.

The adoption of the WHO Pandemic Agreement in May 2025, marked the first formal and explicit inclusion of the One Health approach in a high-level, potentially binding, global health framework. Although elements of One Health were previously present in the International Health Regulations, adopted by the World Health Assembly, this agreement crystallises the concept as a central element, highlighting zoonotic threats, biohazards, and potential threats such as Pathogen X. The inclusion of an article on One Health reflects a shift toward socioecological thinking in global health architecture. The ratification of the Agreement is expected by 2027 at the earliest, but it faces a fragile geopolitical landscape. Concerns over national sovereignty remain a key point of resistance, despite clear language ruling out the WHO authority to enforce or prescribe national policies. Structurally, the Agreement is a broad framework of guiding principles rather than a directly transformative tool. Its success will largely depend not on enforcement, but on soft power, trust, and long-term cooperation, much like the case with the International Health Regulations. Countries should be motivated to follow it because it ultimately protects their own health security.

The success of One Health will also depend on addressing the destabilising effects of war and militarisation, which simultaneously devastate ecosystems and weaken already fragile health systems. Armed conflicts continue to be a means of capturing and redistributing increasingly scarce global resources. The humanitarian consequences are profound: wars decimate health-care infrastructure, displace populations, and accelerate disease outbreaks, from cholera to mpox. The ecological toll is equally severe: damaged ecosystems, contaminated water sources, and disrupted food systems further undermine population health. Militarisation diverts essential funding; a study found that a 1.0% increase in military spending is associated with a 0.6% decrease in health spending, and nearly 1.0% in lower-income countries. Redirecting even a fraction of military budgets could substantially strengthen global health and ecological resilience. A pivot toward non-interventionist diplomacy and sustainable cooperation could be a viable alternative, reducing reliance on militarisation and opening fiscal space for health and environmental priorities.

The next pandemic is likely to be zoonotic. WHO's 2024 prioritisation framework confirms that most high-risk pathogens, including the hypothetical Pathogen X, are zoonoses circulating within the human–animal–environment interface. This zoonotic nature complicates the control and prevention of these diseases, as it requires managing both human and animal health. Risks to biosecurity in an increasingly polarised world require effective surveillance and control mechanisms. This reinforces the need to embed the One Health approach across all policies; not just as a concept, but through operational, accountable systems. However, without transparency and equity, even scientifically sound frameworks risk misuse. The unethical use of digital technologies could threaten autonomy, data sovereignty, and civil liberties, particularly in weaker governance contexts. Global health security should not become a pretext for consolidating control or bypassing democratic oversight. The new global health architecture grounded in a socioecological transformation should prioritise equity by protecting the rights and interests of poorer nations and populations, while limiting the dominance of corporate or geopolitical agendas. The growth-dependent model underpinning global health architecture has reached its limits; what follows must be scientifically grounded, structurally fair, and ethically defensible.

